# Preparation and Superstrong Adsorption of a Novel La(Ⅲ)-Crosslinked Alginate/Modified Diatomite Macroparticle Composite for Anionic Dyes Removal from Aqueous Solutions

**DOI:** 10.3390/gels8120810

**Published:** 2022-12-10

**Authors:** Yuting Zhao, Beigang Li

**Affiliations:** 1 Chemistry and Environment Science College, Inner Mongolia Normal University, Hohhot 010022, China; 2 Inner Mongolia Key Laboratory of Environmental Chemistry, Hohhot 010022, China

**Keywords:** adsorption, diatomite, CPAM, sodium alginate, La(III) ions

## Abstract

In order to solve the problem of dye pollution of the water environment, a green macroparticle composite (CPAM-Dia/SA-La) as a bioadsorbent was prepared through a sodium alginate (SA) reaction with a polyacrylamide (CPAM)-modified diatomite (Dia) and further La(III) ion crosslinking polymerization, and characterized by various analytical methods. The important preparation and adsorption conditions of the composite were explored by the adsorption of Acid blue 113 (AB 113) and Congo red (CR) dyes. The dye adsorption efficiency was evaluated. The results show that CPAM-Dia/SA-La composite prepared under the optimized conditions displays superstrong adsorption capacities of 2907 and 1578 mg/g for AB 113 and CR and almost 100% removal efficiency within 60 min adsorption time at pH 2.0 and 298 K, and they decrease slightly with the pH increase to 10. The fitting of equilibrium data to the Langmuir model is the best and the adsorption kinetic processes can be expressed by the Pseudo-second-order kinetic model. The adsorption processes are both spontaneous and exothermic. The analysis results of FT−IR and XPS revealed that the superstrong adsorption of CPAM-Dia/SA-La for dyes. The composite adsorbed by the dye can be recycled. CPAM-Dia/SA-La is a promising biosorbent for dye wastewater treatment.

## 1. Introduction

The dyes industry is a traditional advantageous industry in China and an important industry that concerns the basic livelihood of the people. The dyestuff industry involves various fields, such as textile printing and dyeing, papermaking, rubber, plastics and leather, and has been gradually penetrating into modern high-tech fields including information technology, biotechnology, medical technology and so on. In the revision of the ISO standards for the dyestuff industry, the dyes used are grouped according to the dye standard under ISO/TC256, in accordance with the classification of the technical committees in ISO/IEC, which clearly describes the dyestuff production regulations. Different types of dyes including Acid blue 113 (AB 113) and Congo red (CR) anionic dyes belonging to azo dyes are widely used in production processes due to their bright color, good water solubility and simple application technology [1]. However, a small part of these dyes during production and use are inevitably discharged into water. The azo bonds (−N=N−) contained in these molecules are easily decomposed and reduced to produce a variety of carcinogenic aromatic amines under certain conditions, which not only harm human health, but also cause serious environmental pollution [2]. Therefore, dye wastewater must be effectively purified before discharge. Exploring and developing simple, economical and efficient treatment technologies for dye wastewater have become a research focus [2,3]. So far, many methods such as electrochemical, photocatalytic, biological oxidation, adsorption and other technologies have been used for the purification of dye effluent [3,4,5,6]. Among them, the adsorption method has become one of the most popular methods to treat dye wastewater because of its simple operation, low cost and strong adaptability. Therefore, the development of macroparticle adsorption materials with environmental friendliness, high adsorption capacity, convenient and practical operation and recyclability has attracted a lot of attention [7,8,9,10,11,12,13].

Diatomite (Dia) with rich source is a kind of powdery non-metallic mineral gradually formed by the deposition of the remains of diatoms in the seas or lakes under the action of the natural environment, and its main component is SiO_2_. Dia is mainly composed of the walls and shells of diatoms with the different microporous morphological characteristics of multi-level, large number and orderly arrangement, and has abundant porosity, large specific surface area and good chemical stability. Hence, it has often been used for the adsorption of pollutants in wastewater. However, there are some deficiencies in the use of powder Dia, such as low adsorption capacity, easy loss and difficult separation and recovery. Consequently, Dia has often been modified by different modification methods or combined with other substances to prepare composite materials with higher adsorption properties [14,15,16]. Cationic polyacrylamide (CPAM) is a linear organic polymer that is often used as a flocculant in wastewater treatment [17]. The adsorbent materials prepared by modifying diatomite with CPAM can better adsorb dye molecules in wastewater through physical-chemical interaction and bridging action [18]. Sodium alginate (SA) is a natural polysaccharide polymer with good biocompatibility, non-toxicity and degradability. The large number of hydroxyl and carboxyl groups in alginate molecules can be cross-linked by multivalent metal ions to form macroparticle gel spheres with an “egg box structure” model [19]. These macroparticle gel spheres can be used as an ideal framework for further preparation of gel composites with higher adsorption performance, and related studies have achieved some good results [20,21,22,23]. Rare earth (RE) ions have a unique electronic shell structure and high positive charge. La^3+^, Ce^3+^ and Y^3+^ ions can be used to crosslink SA well to form gel spheres and the relevant research has been reported [24,25,26]. However, gel spheres obtained by mono-ionic polymerization have problems of compactness and insufficient mechanical strength, which are unfavorable to adsorption. Hence, SA has been often used to combine with other materials to synthesize macroparticle adsorption materials with better performance, which can develop their strengths and circumvent their weaknesses and better play their synergistic role [27,28,29,30,31].

Based on the above analysis and previous research foundation, CPAM-Dia modified by CPAM and SA were polymerized by crosslinking La(III) ions using drop polymerization, and the prepared CPAM-Dia/SA-La gel spheres were characterized and used for the removal of anionic dyes in water in this study. The preparation conditions of the gel composite and their adsorption conditions for AB 113 and CR dyes were investigated in detail. The adsorption performance, reusability and adsorption mechanism of CPAM-Dia/SA-La gel spheres would be evaluated and explored separately. We hope to prepare a cost-effective and environmentally friendly composite material with higher adsorption capacity by utilizing natural biological resources and simple and feasible methods to achieve ultra-efficient purification of high-concentration anionic dye wastewater.

## 2. Results and Discussion

### 2.1. Selection of Preparation Conditions of the Gel Composite

It is very important for the composite preparation to establish the optimal process parameters and effective ratio among the raw materials and reagents. Therefore, based on the adsorption test for AB 113 dye, the important preparation conditions of the CPAM-Dia/SA-La gel composite were explored in detail.

**Figure 1:**
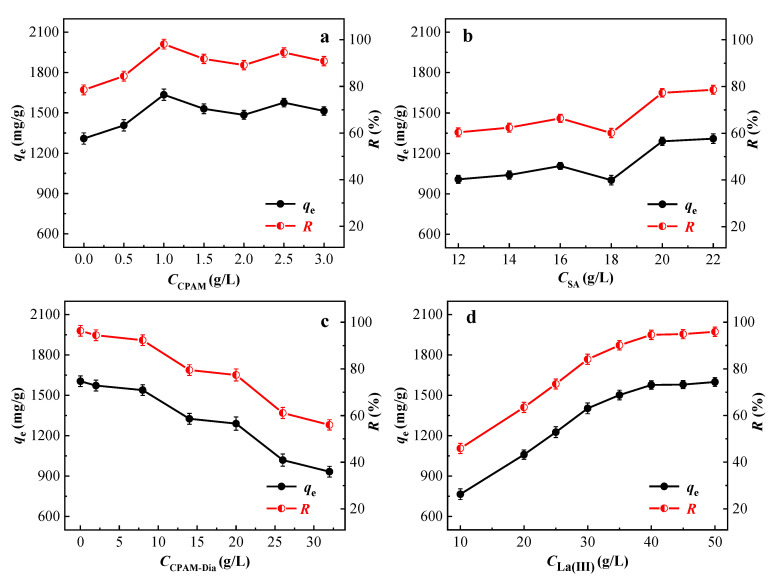
Effect of the concentration of CPAM (**a**), SA (**b**), CPAM-Dia (**c**) and La(Ⅲ) (**d**) on the adsorption property of the gel spheres (2000 mg/L Dye solution 25 mL).

In order to improve the adsorption performance of Dia, CPAM-Dia was prepared by modifying Dia using CPAM with different concentrations, and the adsorption of the obtained CPAM-Dia/SA-La gel composites for AB 113 dye is presented in Figure 1a. The values of the adsorption amount (*q*_e_, mg/g) and removal rate (*R*, %) fluctuated with the increasing of CPAM concentration from 0 to 3 g/L, but the best adsorption effect was achieved when the CPAM concentration was 1 g/L. Therefore, the 1 g/L CPAM solution was selected to prepare gel composite.

The SA concentration is the key to preparing the high-performance gel composite. Hence, the effect of SA concentration on the gel sphere property was further investigated. It can be seen from Figure 1b that the *q*_e_ (mg/g) and *R* (%) values of AB 113 onto the prepared gel spheres gradually increased with the increase of the SA concentration. Dynamic equilibrium can be reached when the SA concentration is 20 g/L, and the commensurable *q*_e_ (mg/g) and *R* (%) values are 1290 mg/g and 77.3%, respectively. After that, the *q*_e_ (mg/g) and *R* (%) values of AB 113 have almost remained unchanged with the increasing the concentration. Therefore, 20 g/L concentration of SA solution was selected for the preparation of CPAM-Dia/SA-La gel composite.

It can be seen in Figure 1c that the *q*_e_ (mg/g) and *R* (%) values of the synthesized CPAM-Dia/SA-La gel spheres for AB 113 slightly decreased from 1605 to 1539 mg/g and 96.3 to 92.3%, respectively, as the concentration of CPAM-Dia increased from 0 to 8 g/L, then continued to decrease with the increase of CPAM-Dia concentration. Meanwhile a gradual increase in gel sphere size could be observed. Considering the effects of production cost and pelleting effect, the CPAM-Dia concentration of 8 g/L was chosen.

When the concentrations of CPAM-Dia and SA solutions were fixed at 8 and 20 g/L, respectively, the effect of the added La(III) ion concentration on the adsorption performance of CPAM-Dia/SA-La gel spheres for AB 113 was investigated. As shown in Figure 1d, when the La(III) ion concentration increased from 10 to 40 g/L, the *q*_e_ (mg/g) and *R* (%) values increased significantly from 765 to 1577 mg/g and 45.9 to 94.6%, respectively, and then changed very little with the continuous increase of La(III) ion concentration. Considering the factors of resource saving, cost reduction and treatment effect, the 40 g/L concentration of La(III) ion solution was selected. The prepared CPAM-Dia/SA-La gel spheres not only have good spheroidization and strong adsorption property, but also overcome the problems of difficult recovery and low adsorption capacity for the pollutant removal in aqueous solutions by Dia.

### 2.2. Characterization of Materials

**Figure 2:**
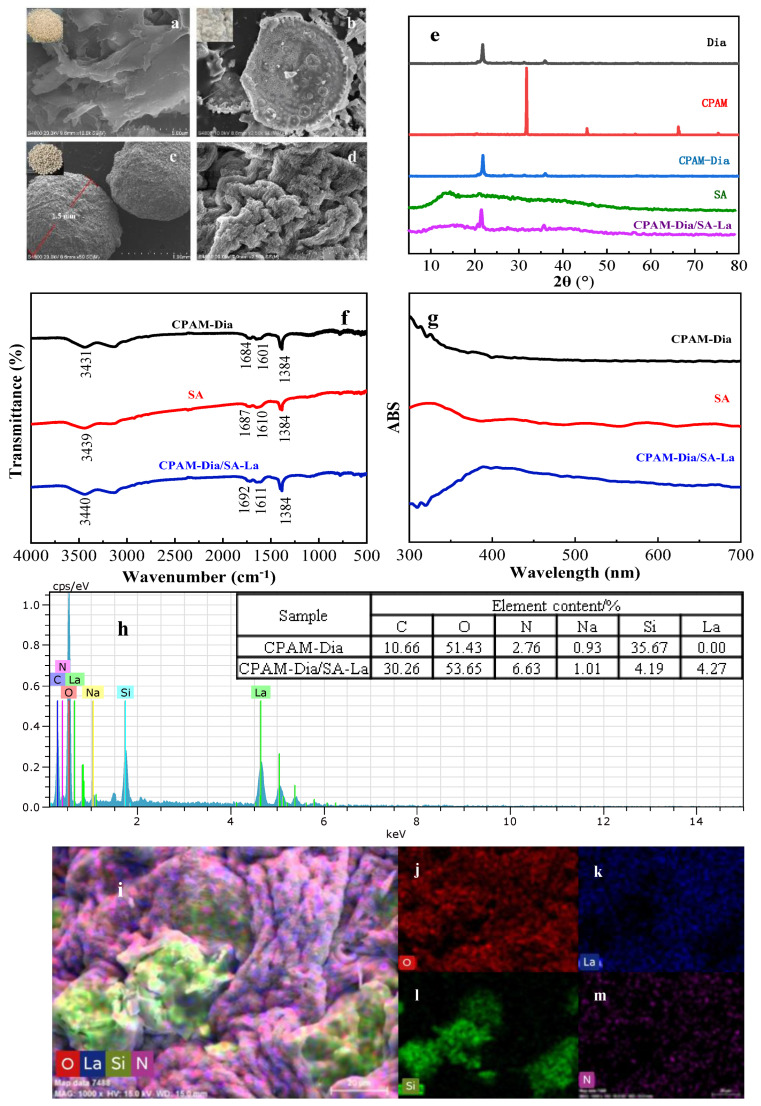
SEM images of SA (**a**), Dia (**b**) and gel spheres (**c**,**d**); XRD (**e**), FT−IR (**f**) and UV−Vis (**g**) spectra; EDS results of raw materials and gel spheres (**h**); Element distribution of the gel composite (**i**–**m**).

The surface microstructure of SA, Dia and CPAM-Dia/SA-La polymer gel spheres were characterized by scanning electron microscopy (SEM). As seen in Figure 2a, the milky-white powder SA consists primarily of some massive and flaky particles. The microscopic morphology of the white powder Dia is a honeycomb-shaped circular flake with dense and uniform distribution of micro-internal pores in Figure 2b. As shown in Figure 2c, the white synthesized particles CPAM-Dia/SA-La are spherical with a diameter of about 1.5 mm, and their surfaces are in the shape of a “cauliflower” (Figure 2d) which is composed of irregular grooves and reticulated folds of varying depths. This structure was extremely conducive to absorbing pollutants [32,33].

In the XRD pattern of Figure 2e, the three diffraction peaks (33.4°, 45.2° and 67.1°) of CPAM almost disappear in the XRD pattern of CPAM-Dia, but the characteristic diffraction peaks of Dia at 2θ = 21.9° and 36.1° are retained, indicating that the reaction between CPAM and Dia. In the XRD pattern of CPAM-Dia/SA-La, the distinctive diffraction peaks at 2θ = 13.2°, 21.3° and 37.2° of SA mostly vanished, and the major diffraction peaks at 2θ = 21.9° and 36.1° of Dia are still visible [30], indicating the successful synthesis of CPAM-Dia/SA-La gel spheres.

In the CPAM-Dia FT−IR spectrum of Figure 2f, the absorption bands at 3431, 1684 and 1384 cm**^−^**^1^ are stretching vibration peaks of −OH, C=O and C−O, respectively, while the peak at 1601 cm**^−^**^1^ is bending the vibration peak of N-H [31,34]. The absorption bands at 3439, 1687, 1610 and 1384 cm**^−^**^1^ in the SA spectrum correspond to the −OH and C=O stretching vibration peaks and asymmetric and symmetric stretching vibration peaks of −COO^−^, respectively. In the CPAM-Dia/SA-La spectrum, the stretching vibration peaks of −OH and C=O and the asymmetric stretching vibration peak of −COO^−^ were shifted to 3440, 1692 and 1611 cm^−1^, respectively, while the symmetric stretching vibration peak of −COO^−^ became larger in the peak area. This is because the O−containing active groups on the composite participated in the interaction with dye anions [35], showing the successful synthesis of the CPAM-Dia/SA-La composite.

The UV-vis analysis results for CPAM-Dia, SA, and CPAM-Dia/SA-La are depicted in Figure 2g. CPAM-Dia has no significant characteristic absorption peak between 300 and 700 nm, and an absorption peak at 425 nm appears in the SA spectrum. However, a new absorption peak in the shape of steamed bread appears in the CPAM-Dia/SA-La spectrum. This is due to due to the interaction between SA and CPAM-Dia through the cross-linking polymerization of La(III) ions, forming a novel macroparticle gel composite that can be seen by the naked eyes [24].

After further EDS analysis of the samples (see Figure 2h), compared with the element distribution of CPAM-Dia, a La element with a content of 4.27% appeared in the CPAM-Dia/SA-La gel spheres, while the content of the Si element decreased from 35.67 to 4.19%, further showing that the La(III) ions in solution underwent a cross-linking polymerization reaction with SA to form a CPAM-Dia/SA-La gel composite with a three-dimensional network structure [35]. The surface scan analysis of CPAM-Dia/SA-La (Figure 2i–m) reveals that the O, La, Si, and N elements are evenly distributed in the composite, indicating the successful preparation of the target composite.

### 2.3. Adsorption Research 

#### 2.3.1. Research on Important Adsorption Conditions

**Figure 3:**
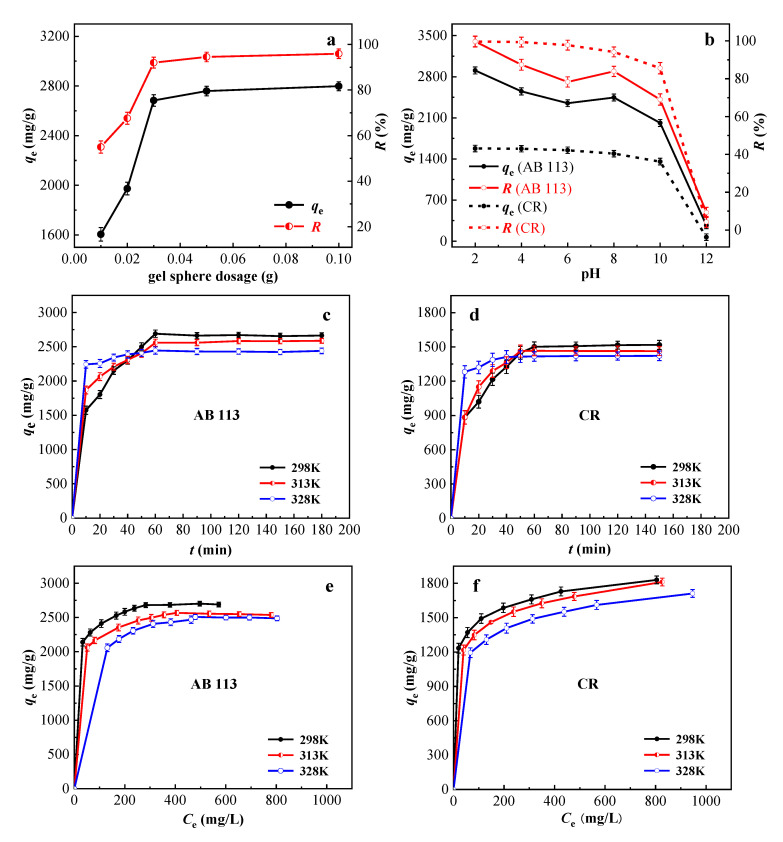
Effect of gel sphere dosage (**a**), pH (**b**), contact time and temperature on adsorption (**c**,**d**); isothermal adsorption curves (**e**,**f**).

Determining the optimal dosage of CPAM-Dia/SA-La gel spheres can make more efficient and rational use of resources. It can be seen from Figure 3a that the *q*_e_ (mg/g) and *R* (%) values of the adsorbent to AB 113 increased from 1605 to 2683 mg/g and 55.0 to 92.0%, respectively, as the dosage of CPAM-Dia/SA-La was increased from 0.01 to 0.03 g. That is because increasing the adsorbent dosage provides more adsorption sites for the dye ions. The *q*_e_ (mg/g) and *R* (%) values of the adsorbent for the dye remained almost unchanged when the adsorbent dosage was continuously increased to 0.1 g. This is due to the constant concentration of adsorbate. The amount of dye adsorbed did not change even if the adsorbent dosage increased. Considering things comprehensively, the adsorbent dosage of 0.03 g was chosen.

The effect of pH on the adsorption of AB 113 and CR onto CPAM-Dia/SA-La was examined, as shown in Figure 3b. The *q*_e_ (mg/g) and *R* (%) values of AB 113 and CR by the composite reached the maximum of 2907 and 1578 mg/g, 99.7 and 99.7%, respectively at pH 2.0, and could still reach 2013 and 881 mg/g, 69.0 and 85.6% with the increase of pH to 10.0, and then decreased rapidly with the continuous increase of pH. The measured zero charge point (pH_pzc_) of CPAM-Dia/SA-La gel composite is 6.26. When the solution pH is less than pH_pzc_, the protonation of CPAM-Dia/SA-La gel spheres caused strong electrostatic adsorption between the adsorbent and dye anions, leading to a significant increase of the *q*_e_ and *R* values. When the solution pH was greater than pH_pzc_ value, the *q*_e_ (mg/g) and *R* (%) values should decrease significantly with further increase of pH due to the electrostatic repulsion between the adsorbent with negative surface charge and the dye anions. However, the experimental results were not the case when the solution pH is equal to or less than 10 (Figure 3b). This should be due to various hydrogen bonding between gel spheres with abundant −COOH and −OH groups and dye anions with −N=N−, −NH, −OH and aromatic rings and possible complexation between unsaturated La(III) on the surface of gel spheres and reactive functional groups in dye anions [25,35,36,37]. The *q*_e_ (mg/g) and *R* (%) values of CPAM-Dia/SA-La gel spheres for AB 113 and CR were, respectively 2696 and 1477 mg/g, 92.4 and 93.3% under natural pH (7.08 and 9.94, respectively) of dye solutions. The results show that CPAM-Dia/SA-La gel composite exhibits the superhigh adsorption capacities and almost 100% removal efficiency for two anionic dyes in a wide pH range from 2.0 to 10.0.

The effects of different adsorption time and temperatures on the adsorbent performance were further examined. As depicted in Figure 3c,d, the adsorption amounts of CPAM-Dia/SA-La for AB 113 and CR grew dramatically within 30 min at 298 K, and then increased slowly with increasing time. The equilibrium adsorption amounts reach 2690 and 1501 mg/g, respectively for AB 113 and CR by adsorbent within 60 min. This is because as the adsorption time increases, a large number of adsorption sites on the surface of the gel spheres are gradually covered by dye molecules, eventually reaching dynamic adsorption equilibrium. Furthermore, the adsorption rate increases with increasing temperature before reaching the adsorption equilibrium, but decreases with increasing temperature after the adsorption equilibrium, illustrating that the temperature did not affect the time required for adsorption to reach equilibrium (Figure 3c,d). It shows that the adsorption of the two dyes by the gel spheres was exothermic. The best results for the decolorization of anionic dye wastewater were obtained at 298 K.

#### 2.3.2. Study on Adsorption Kinetics

In order to further explore the adsorption process mechanism of CPAM-Dia/SA-La for AB 113 and CR dyes, the adsorption kinetic data at different temperatures were fitted by Pseudo-first-order and Pseudo-second-order adsorption rate Equations (1) and (2), respectively.

ln(*q*_e_ − *q*_t_) = ln*q*_e_ − *k*_1_*t* (1)

*t*⁄*q*_1_ = 1⁄(*k*_2_*q*_e_^2^) + *t*⁄*q*_e_ (2)

where *q*_e_ and *q*_t_ (mg/g) are the adsorption capacities at adsorption equilibrium and adsorption time *t* (min), respectively; *k*_1_ (min^−1^) and *k*_2_ [g/(mg·min)] are the adsorption rate constants of Pseudo-first-order and Pseudo-second-order adsorption models, respectively. The fitting parameters obtained are shown in Table 1.

It can be seen from Table 1, by comparing the correlation coefficient (*R*^2^) of the two models, the kinetic data are more consistent with the Pseudo-second-order model (*R*^2^ ≥ 0.997) and had a more perfect goodness-of-fit (*RMSE* ≤ 4.1 × 10^−6^) for the Pseudo-second-order model than the Pseudo-first-order model (*RMSE* ≤ 2.9 × 10^−2^). The equilibrium adsorption capacities (*q*_e,c_, mg/g) obtained by the Pseudo-second-order adsorption rate equation were extremely close to the actual equilibrium adsorption capacities (*q*_e,exp_, mg/g) for two dyes, showing that the entire adsorption processes of CPAM-Dia/SA-La gel spheres for two anionic dyes at different temperatures can be accurately described by the Pseudo-second-order kinetic model. In addition, the Pseudo-second-order rate constant (*k*_2_) decreases with increasing temperature after the adsorption equilibrium, indicating that this adsorption is exothermic in nature.

#### 2.3.3. Study on Adsorption Isotherms and Adsorption Thermodynamics

The adsorption isotherms of CPAM-Dia/SA-La at various temperatures for AB 113 and CR are shown in Figure 3e,f. As the equilibrium dye concentration *C*_e_ (mg/g) increased, the adsorption capacity *q*_e_ (mg/g) increased, while the *R* (%) value decreased slightly. The adsorption capacity of the adsorbent decreased with the increase of temperature, which is consistent with the effect of temperature on sorption kinetics. In order to better describe the adsorption behavior, Langmuir and Freundlich isotherm adsorption models were used to fit the isothermal adsorption data of two dyes on CPAM-Dia/SA-La gel spheres. The linear expressions of the Langmuir model and the Freundlich model are Equations (3) and (4), respectively:

*C*_e_⁄*q*_e_ = *C*_e_⁄*q*_m_ + 1⁄(*q*_m_*K*_L_) (3)

ln*q*_e_ = (1⁄*n*)ln*C*_e_ + ln*K*_F_ (4)

where *C*_e_ (mg/L) is the equilibrium concentration of dye solution; *q*_e_ (mg/g) is the adsorption capacity at equilibrium; *q*_m_ (mg/g) is the monolayer-saturated adsorption capacity; *K*_L_ (L/mg) is Langmuir adsorption coefficient; *K*_F_ and *n* are the Freundlich empirical constants.

The results in Table 2 show that the *R*^2^ values of the correlation coefficients obtained by fitting the experimental data using the Langmuir isothermal model at different temperatures (*R*^2^ ≥ 0.998) are closer to 1.000, and the fitting results at each temperature (*RMSE* ≤ 5.6 × 10^−5^) is significantly better than those by the Freundlich model (*RMSE* ≤ 7.5 × 10^−4^). The saturated adsorption capacities *q*_m_ (mg/g) for the dyes were very close to the actual equilibrium values (*q*_e,exp_, mg/g) (Table 1). The CPAM-Dia/SA-La gel composite is an inorganic/organic polymer with a unique surface and three-dimensional net-like structure (see Figure 2c,d), and abundant O−containing and N−containing groups and unsaturated La(III) on its surface have strong interactions with dye molecules with different groups, including hydrogen bonding, complexation and electrostatic adsorption. Therefore, gel spheres display high adsorption capacities for anionic dyes. The Langmuir adsorption coefficient (*K*_L_) decreased progressively with increasing temperature, which indicates that the adsorption of two anionic dyes by CPAM-Dia/SA-La gel spheres was exothermic, which is consistent with the results on the effect of temperature on kinetics. 1/*n* is less than 1, indicating that the adsorption process of each dye is relatively easy to carry out.

Thermodynamic parameters such as Gibbs free energy change (Δ*G*, kJ/mol), entropy change (Δ*S*, J/(mol·K)) and enthalpy change (Δ*H*, kJ/mol) can be calculated by Equations (5) and (6).

Δ*G* = −*RT*ln*k* (5)

Δ*G* = Δ*H* − *T*Δ*S* (6)

where *R* is the thermodynamic characteristic constant, 8.314 J/(mol·K); *T* (K) is the absolute temperature and *k* (*k* = *q*_e_/*C*_e_) (L/g) is the adsorption equilibrium constant. The slope and the intercept of the straight line obtained by plotting Δ*G* against *T* are the values of Δ*S* and Δ*H*. 

Thermodynamic parameters obtained from calculation are given in Table 2. According to the results in Table 2, Δ*G* < 0 indicates that the adsorption can occur spontaneously. Δ*H* < 0 indicates that the adsorption of CPAM-Dia/SA-La for both dyes is exothermic, which is consistent with the previous studies. Δ*S* > 0 indicates that the adsorption of each dye is an entropy-increasing process [38]. In general, |Δ*H*| values between 2~40 kJ/mol represent the H−bonding force, Δ*G* values between −20~0 kJ/mol illustrate physical adsorption and Δ*G* values between −80~−400 kJ/mol represent chemical adsorption [39]. In this study, the Δ*H* values ranging from −15.7 to −19.1 kJ/mol show the presence of hydrogen bonds in the interaction between CPAM-Dia/SA-La and dye anions. The Δ*G* values between −26.3~−32.9 kJ/mol suggest that physical and chemical adsorption coexisted in the CPAM-Dia/SA-La adsorption on both dyes. 

The *q*_max_ (mg/g) values of AB 113 and CR onto other adsorbents reported are shown in Table 3 [40,41,42,43,44,45,46,47,48,49,50,51,52,53,54,55,56,57,58,59,60]. Through comparison, it is found that CPAM-Dia/SA-La has more significant adsorption advantages for two dyes than other adsorption materials. CPAM-Dia/SA-La not only has ultra-high adsorption capacities, but also is very easy to separate and recover from water without secondary pollution as a macroparticle adsorbent. Therefore, CPAM-Dia/SA-La gel spheres are a highly prospective greener adsorbent expected to be used to treat practical anionic dye wastewater effectively. 

#### 2.3.4. Comparison of Adsorption Properties of Various Materials

**Figure 4:**
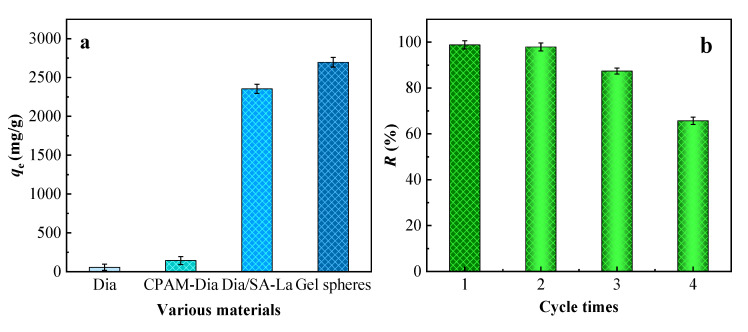
Comparison of adsorption properties of different materials (**a**); Effect of regeneration cycle times on regeneration rate (**b**).

The adsorption effects of Dia, CPAM-Dia, Dia/SA-La and CPAM-Dia/SA-La for AB 113 according to the adsorption test method are shown in Figure 4a. The adsorption capacity of a single Dia for AB 113 was only 56 mg/g, but the adsorption capacity of AB 113 by CPAM-Dia modified by CPAM was increased to 143 mg/g, which is about 2.6 times higher that of Dia. The adsorption capacities of Dia/SA-La and CPAM-Dia/SA-La for AB 113 have significantly increased to 2354 and 2696 mg/g, which are 42 and 48 times those of Dia for AB113, respectively. The modification of Dia and further preparation of inorganic/organic macroparticle gel composite significantly improved the adsorption performance of Dia and overcame the shortcomings of Dia powder which was easy to lose and difficult to recover in the water. Hence, CPAM-Dia/SA-La gel spheres have the benefits of green environmental protection, superstrong adsorption capacity, good selectivity and easy separation from the water and without secondary pollution. At the same time, the *q*_max_ (mg/g) values of other Dia-based adsorbents for the dyes are shown in Table 4 [61,62,63,64,65,66]. By comparing the reported research results, the CPAM-Dia/SA-La gel composite is confirmed to have extremely significant adsorption advantages for dyes.

#### 2.3.5. Regeneration Study

According to the regeneration experimental method, different regenerants such as Ethylene Diamine Tetraacetic Acid (EDTA), HCl, NaOH, Tartaric Acid (TA), La(III), Fe(III) and Al(III) solutions were used for the desorption treatment of dye-adsorbed CPAM-Dia/SA-La, respectively. The experimental results indicate that the combination of EDTA and La(III) solutions has the most significant regeneration effect, as depicted in Figure 4b. After adsorption and desorption of AB 113 by CPAM-Dia/SA-La gel spheres, the regeneration rates after three cycles of dye adsorption/desorption were 99.7%, 88.5% and 66.5%, respectively. This shows that CPAM-Dia/SA-La composite can be reused at least three times and is an effective and renewable macroparticle adsorbent.

#### 2.3.6. Discussion on the Adsorption Mechanism

**Figure 5:**
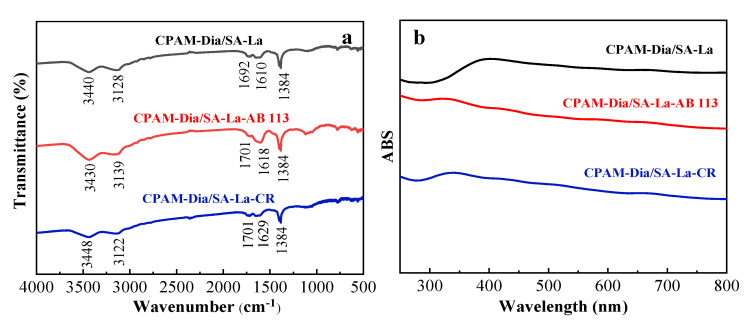
FT−IR (**a**) and UV−Vis (**b**) spectra of CPAM-Dia/SA-La before and after dye adsorption.

The FT−IR spectra of CPAM-Dia/SA-La gel spheres before and after the adsorption of AB 113 and CR as shown in Figure 5a. The asymmetric stretching vibration absorption peaks of −COO^−^ in the CPAM-Dia/SA-La FT−IR spectrum were displaced from 1610 to 1618 cm^−1^ and 1629 cm^−1^ after AB 113 and CR adsorption in the CPAM-Dia/SA-La-AB 113 and CPAM-Dia/SA-La-CR FT−IR spectra, respectively, while the stretching vibration absorption peaks of C=O shifted from 1692 cm^−1^ to 1701 and 1701 cm^−1^, respectively. The intensity of the −COO^−^ symmetric vibration peak at 1384 cm^−1^ also changed [35], this may be caused by H−bonding between the adsorbent and the dye molecules. This indicates that the chemical reaction occurred in the adsorption process of each dye.

The UV−vis absorption spectra of the CPAM-Dia/SA-La composite were determined before and after the adsorption of two dyes. It can be seen in Figure 5b, there is a clear absorption peak with bread-steamed shape at 400 nm in the CPAM-Dia/SA-La spectrum. The absorption peaks were moved to 320 and 340 nm, respectively, and the shape of two peaks also changed significantly in the absorption spectra of CPAM-Dia/SA-La-AB 113 and CPAM-Dia/SA-La-CR. This indicates that the adsorbent interacts chemically with the dye molecules.

**Figure 6:**
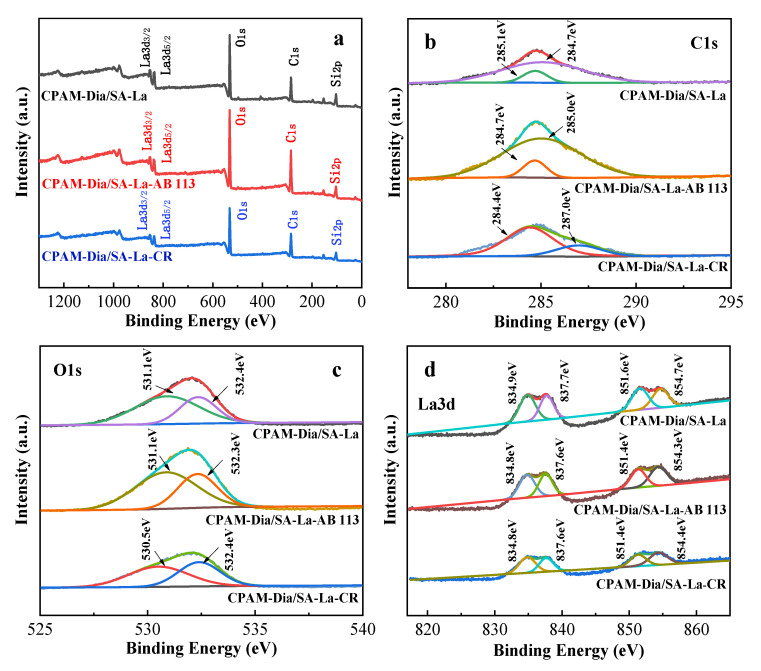
XPS spectra of the gel composite before and after dye adsorption (**a**–**d**).

It can be seen in Figure 6 the XPS wide spectrum scan findings and the fine scan high-resolution spectra of C 1s, O 1s and La 3d before and after dye adsorption by CPAM-Dia/SA-La. As shown in Figure 6a, the peak intensities of O, C, La and Si in the CPAM-Dia/SA-La XPS spectrum changed significantly after dye adsorption, showing interaction between the adsorbent and the dye molecules. In the high-resolution XPS spectrum of C 1s (Figure 6b), the two peaks at binding energies of 285.1 (C−O, C−OH) and 284.7 eV (C−O−C) were shifted to 284.7 and 285.0 eV after AB 113 adsorption, and 284.4 and 287.0 eV after CR adsorption, respectively. Furthermore, the corresponding peak area ratios became 0.6:1 and 1.1:1 before and after adsorption of AB 113 and CR, respectively [31,32]. Two peaks at binding energies of 531.1 (C−OH, C−O−C) and 532.4 eV (COO^−^) on the O 1s spectrum of the composite (Figure 6c) were moved to 532.3 eV for AB 113 and 530.5 eV for CR after adsorption, and the peak area ratios decreased to 0.8:1 and 0.6:1, respectively [36,38,67,68]. The four peaks belonging to La 3d_5/2_ and La 3d_3/2_ at 834.9 and 837.7 eV, 851.6 and 854.7 eV in the high-resolution XPS spectra of La 3d (Figure 6d) shifted slightly toward the low-field and the peak area ratios reduced to 0.9:1 and 0.6:1 after dye adsorption, respectively. The above changes in binding energy and peak area further suggest that the various O−containing active groups and La(III) ions in the CPAM-Dia/SA-La gel spheres have chemical and hydrogen bond interactions with −N=N−, −NH_2_, −OH and aromatic rings in dye molecules [35], which is also consistent with the FT−IR and UV−vis analytical results.

**Figure 7:**
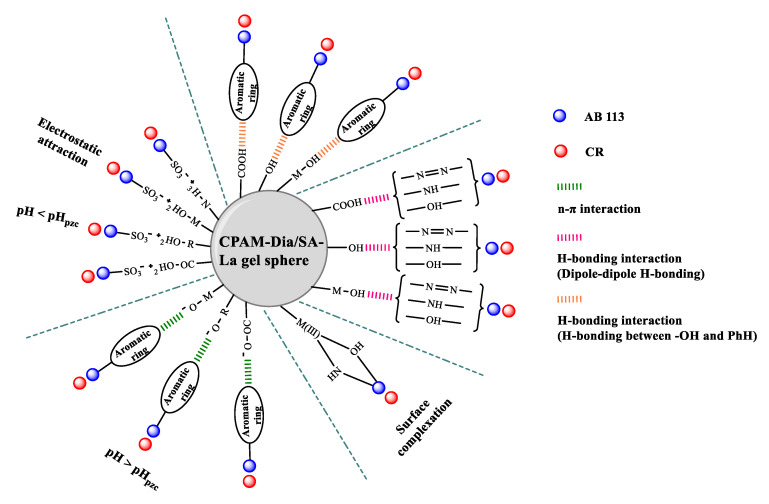
Interaction sketch between the gel composite and anionic dyes.

The research results of pH effect, FT−IR, UV−vis and XPS characterization, adsorption kinetics and thermodynamics sufficiently demonstrate that there are various interactions between CPAM-Dia/SA-La and dye molecules, mainly including electrostatic attraction, complexation reaction and various H−bonding (Figure 7), which can effectively promote the strong multipoint adsorption of CPAM-Dia/SA-La for anionic dyes. Therefore, the gel composite exhibits superhigh adsorption capacities for anionic dyes.

## 3. Conclusions

The CPAM-Dia/SA-La gel composite synthesized by the droplet polymerization method is a “cauliflower”-shaped macroparticle polymer and can be directly used for the effective removal of anionic dyes from wastewater. The adsorption capacities and removal rates of the gel composite for AB 113 and CR can reach 2907 and 1578 mg/g, 99.7 and 99.7% at pH 2.0 and 298 K, respectively, and decrease slightly with pH increasing to 10.0. CPAM-Dia/SA-La macroparticle gel spheres have great adsorption advantages with fast adsorption rate, short equilibrium time, wide pH application range and easy separation and recovery, and can be reused at least three times. The Pseudo-second-order rate model and Langmuir model can accurately describe the spontaneous adsorption processes with exothermic properties and dye adsorption behavior. As an eco-friendly and high-value-added macroparticle composite, CPAM-Dia/SA-La gel spheres will have a very good application potential for treating anionic dye-containing wastewater. 

## 4. Materials and Methods

### 4.1. Materials and Reagents

**Figure 8:**
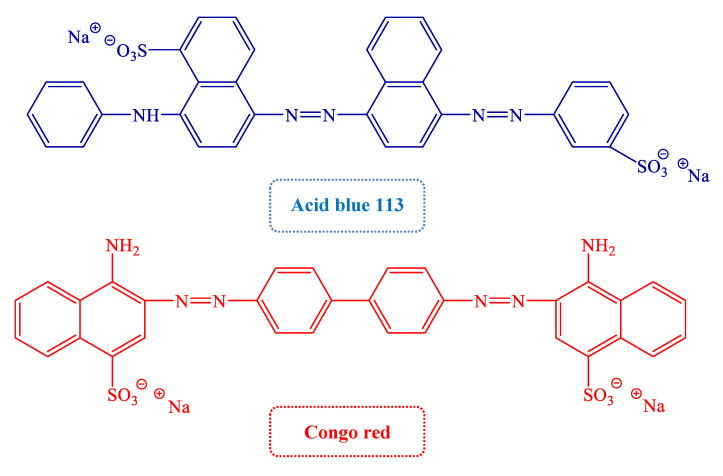
Molecular Structures of AB 113 and CR dyes.

The raw materials and reagents mainly include: Sodium Alginate (SA, AR), Diatomite (Dia, AR) and polyacrylamide (CPAM, AR), which were purchased from Damao Chemical Reagent Factory (Tianjin, China); La(NO_3_)_3_∙6H_2_O (AR) was purchased from Shandong West Asia Chemical Co., Ltd. (Shandong, China); the structural formula of Acid blue 113 (AB 113, λ_max_ = 565 nm, Mr = 681.66), and Congo red (CR, λ_max_ = 498 nm, Mr = 696.68) dyes from Jiaying Chemical Co., Ltd. (Shanghai, China) is shown in Figure 8.

### 4.2. Preparation of CPAM-Dia/SA-La Gel Composite

The specific preparation process of the target product was as follows: the mixture of 20 mL CPAM solution with a certain concentration and 6 g Dia was stirred and dried first, and then 8 g/L obtained CPAM-Dia solution was added to 25 mL SA solution of 20 g/L and stirred evenly. The mixed CPAM-Dia-SA solution was dropped into 100 mL La(NO_3_)_3_ solution of 40 g/L using a syringe, and bead-like macroparticle gel spheres formed continuously and were observed in the solution. After the reaction was completed, the gel spheres continued to be cured in solution for 1.5 h, then were removed, washed and dried. The finally dried CPAM-modified diatomite/lanthanum alginate (CPAM-Dia/SA-La) macroparticle gel spheres were then prepared.

### 4.3. Adsorption Experiments

An amount of 0.03 g CPAM-Dia/SA-La gel spheres were added to a series of 25 mL AB 113 and CR solutions, respectively, which were shaken at constant temperature for 60 min and then filtered with a 0.45 μm filter membrane; the filtrate was measured at λ_max_ 565 nm and 498 nm, respectively. The adsorption amount *q*_e_ (mg/g) and removal rate *R* (%) of gel spheres for dyes were calculated according to Equations (7) and (8).

*q*_e_ = (*C*_0_ − *C*_e_) × *V*/*m* (7)

*R* = (*C*_0_ − *C*_e_)/*C*_0_ × 100% (8)

where *V* (L) represents the volume of the dye solution; *m* (g) represents the adsorbent mass; *C*_0_ and *C*_e_ (mg/L), respectively represent the mass concentration of dye solution before and after adsorption.

### 4.4. Characterization of Materials

The main instruments used in the study are as follows: Hitachi s−4800 field emission scanning electron microscope (SEM−EDS) was used to characterize the micro morphology of materials and the content distribution of various elements; the crystal structure of various materials was determined by X−ray Philips pw−1830 diffraction (XRD) using a Cu−Kα radiation source (λ = 0.154056 nm); FT−IR 6700 infrared spectrometer (FT−IR) and U−2900 UV−visible diffuse reflectance spectrometer (UV−vis) were used to study molecular structure and functional group changes; XPS analysis of samples was performed on an X−ray photoelectron spectrometer (XPS) with a monochromatic Al Ka X−ray source. The point of zero charge (pH_pzc_) of the composite was determined according to the method in this literature [67].

### 4.5. Regeneration Experiment

According to the adsorption test method, 0.03 g CPAM-Dia/SA-La was added to 25 mL of 500 mg/L dye solution, vibrated at 25 °C for 60 min, filtered and then dried. The dried dye-adsorbed gel composite was added to a 25 mL EDTA solution with a certain concentration and vibrated for a period of time, and then the gel spheres were removed and added to 25 mL La(III) solution of 50 g/L to soak for a certain time, after which the gel spheres were dried for the next adsorption/desorption cycle test. The regeneration rate (*RR*) of the gel composite after each cycle test was calculated by Equation (9).

*RR* = *q*_en_/*q*_e1_ × 100% (9)

where *q*_e1_ (mg/g) represents the first adsorption amount of the initial adsorbent; *q*_en_ (mg/g) represents the adsorption amount of each regenerated adsorbent for dyes.

## Figures and Tables

**Table 1:** Kinetic fitting relevant parameters of dye adsorption on CPAM-Dia/SA-La.

Dyes	*T*/K	*q*_e,exp_ (mg/g)	Pseudo-First-Order			
			*k*_1_ (min)	*q*_e,c_ (mg/g)	*R* ^2^	*RMSE* × 10^−2^
AB 113	298	2692	0.0215	473	0.375	2.9
	313	2589	0.0350	835	0.931	0.3
	328	2447	0.0157	100	0.386	1.5
CR	298	1520	0.0372	610	0.914	0.5
	313	1468	0.0269	148	0.494	2.8
	328	1424	0.0236	64	0.777	0.6
**Dyes**	***T*** **/K**	***q*** **_e,exp_** **(mg/g)**	**Pseudo-Second-Order**			
			***k*** **_2_** **[g/(mg·min)]**	***q*** **_e,c_** **(mg/g)**	***R*** **^2^**	***RMSE*** **× 10^−6^**
AB 113	298	2692	6.20 × 10^−5^	2768	0.997	1.4
	313	2589	9.60 × 10^−5^	2648	0.999	0.5
	328	2447	5.01 × 10^−4^	2447	1.000	0.1
CR	298	1520	1.06 × 10^−4^	1579	0.997	4.1
	313	1468	1.91 × 10^−4^	1501	0.999	2.3
	328	1424	9.54 × 10^−4^	1429	1.000	0.9

**Table 2:** Fitting results of two isotherm models for equilibrium data and thermodynamic parameters of dye adsorption onto the gel composite.

Dyes	*T*/K	Langmuir			
		*q*_m_ (mg/g)	*K*_L_ (L/mg)	*R* ^2^	*RMSE* × 10^−5^
AB 113	298	2762	0.076	1.000	0.9
	313	2585	0.085	0.999	0.6
	328	2570	0.033	0.999	0.5
CR	298	1863	0.039	0.999	3.9
	313	1863	0.026	0.998	5.5
	328	1779	0.020	0.998	5.6
**Dyes**	***T*** **/K**	**Freundlich**			
		**1/*n***	***K*** **_F_**	***R*** **^2^**	***RMSE*** **× 10^−4^**
AB 113	298	0.0859	1.61 × 10^3^	0.945	4.0
	313	0.0818	1.53 × 10^3^	0.887	7.5
	328	0.1063	1.27 × 10^3^	0.878	6.3
CR	298	0.1082	8.93 × 10^2^	0.999	0.3
	313	0.1302	7.58 × 10^2^	0.999	0.2
	328	0.1372	6.73 × 10^2^	0.999	0.3
**Dyes**	***T*** **/K**	**Thermodynamic parameters**			
		**Δ** ***G*** **(kJ/mol)**		**Δ** ***H*** **(kJ/mol)**	**Δ** ***S*** **[kJ/(mol·K)]**
AB 113	298	−26.3		−15.9	0.035
	313	−26.9			
	328	−27.4			
CR	298	−31.6		−19.1	0.042
	313	−32.2			
	328	−32.9			

**Table 3:** Maximum adsorption capacities of AB 113 and CR on various adsorbents.

Dyes	Adsorbents	*q*_e(max)_ (mg/g)	Ref.
AB 113	IRA 402(Cl^−^)	130	[40]
	AC−ZnO	333.33	[41]
	Overripe Cucumis sativus peel	59.81	[42]
	HAP	153.85	[43]
	MC	295	[44]
	C−Fe_2_O_3_	128	[45]
	ACF/Fe_3_O_4_	121.4	[46]
	CPAM-Dia/SA-La	2907	This study
CR	Rod-like γ-alumina/volcanic rock porous material	243	[47]
	PSI−PA	522.2	[48]
	DOX KCF−100	240	[49]
	CR KCF−100	547	
	MgO/CA	344.8	[50]
	MgO/GO	684.85	[51]
	ZIF−8	1339.8	[52]
	BNNS@Fe_3_O_4_	499	[53]
	CMC−MMT	74.13	[54]
	Al_2_O_3_−ZrO_2_	57.50	[55]
	Fe_3_O_4_@SiO_2_@ZnTDPAT	12.68	[56]
	MgFe_2_O_4_−NH_2_NPs	71.4	[57]
	pTSA doped PANI@graphene oxide	66.66	[58]
	PANI/ZTO	54.51	[59]
	PANI@ZnO	69.82	[60]
	CPAM-Dia/SA-La	1578	This study

**Table 4:** Maximum adsorption capacities of Dia-based adsorbents for dyes.

Adsorbents	Dyes	*q*_e(max)_ (mg/g)	Ref.
LDH/Dia	TR	555.6	[61]
	ER	625.2	
DTA−MTMS	MG	2.147	[62]
Vietnamese Dia	AFDL	528	[63]
Dia	Rh B	17.04	[64]
MCDBS	MB	116.6	[65]
	MV	61.1	
ND	E 120	12	[66]
CPAM-Dia/SA-La	AB 113	2907	This study

## Data Availability

Not applicable.
